# Water-borne pharmaceuticals reduce phenotypic diversity and response capacity of natural phytoplankton communities

**DOI:** 10.1371/journal.pone.0174207

**Published:** 2017-03-22

**Authors:** Francesco Pomati, Jukka Jokela, Sara Castiglioni, Mridul K. Thomas, Luca Nizzetto

**Affiliations:** 1 Eawag: Swiss Federal Institute of Water Science and Technology, Dübendorf, Switzerland; 2 ETH Zurich, Institute for Integrative Biology, Dübendorf, Switzerland; 3 IRCCS – Istituto di Ricerche Farmacologiche “Mario Negri”, Milano, Italy; 4 NIVA: Norwegian Institute for Water Research, Oslo, Norway; 5 RECETOX, Masaryk University, Brno, Czech Republic; Loyola University Chicago, UNITED STATES

## Abstract

Chemical micropollutants occur worldwide in the environment at low concentrations and in complex mixtures, and how they affect the ecology of natural systems is still uncertain. Dynamics of natural communities are driven by the interaction between individual organisms and their growth environment, which is mediated by the organisms’ expressed phenotypic traits. We tested whether exposure to a mixture of 12 pharmaceuticals and personal care products (PPCP) influences phenotypic trait diversity in lake phytoplankton communities and their ability to regulate biomass production to fit environmental changes (response capacity). We exposed natural phytoplankton assemblages to three mixture levels in permeable microcosms maintained at three depths in a eutrophic lake for one week, during which the environmental conditions were fluctuating. We studied individual-level traits, phenotypic diversity and community biomass. PPCP reduced individual-level trait variance and overall community phenotypic diversity, but maintained higher standing phytoplankton biomass compared to untreated controls. Estimated effect sizes of PPCP on traits and community properties were very large (partial Eta-squared > 0.15). The PPCP mixture antagonistically interacted with the natural environmental gradient in habitats offered by different depths and, at concentrations comparable to those in waste-water effluents, prevented communities from converging to the same phenotypic structure and total biomass of unexposed controls. We show that micropollutants can alter individual-level trait diversity of lake phytoplankton communities and therefore their capacity to respond to natural environmental gradients, potentially affecting aquatic ecosystem processes.

## Introduction

The widespread contamination of natural systems with thousands of anthropogenic chemicals is one of the key environmental concerns of our society [1, 2]. Pollution can have severe impacts on local populations in exposed environments, reducing the structural and functional diversity of ecological communities, which supports ecosystem processes and services [3–6]. Biodiversity, including variation among organisms in genes and phenotypes, increases the efficiency of ecosystem functions and stabilises ecosystem processes in a changing environment, thereby determining the resilience of an ecosystem [7–9]. Phenotypic traits, encompassing any expressed morphological, physiological or life-history features of organisms, are therefore a fundamental component of biodiversity, tightly linked to fitness and capturing individual responses to abiotic and biotic environmental factors and community interactions [10–12]. The phenotypic structure and diversity of a community can provide understanding and prediction of ecosystem processes [13], responses to chemical pollutants [5, 14, 15], and of a community’s ability to maintain ecosystem function in the face of environmental changes [7, 16]. We have limited knowledge, however, of how diffuse chemical pollution influences phenotypic diversity and the functioning of natural communities over environmental gradients.

In this study we asked the question of whether a mixture of pharmaceuticals and personal care products (PPCP) can affect phenotypic diversity measured with individual-level morphological data in phytoplankton, and consequently reduce the capacity of a community to adapt structure (e.g. trait composition) and functioning (e.g. production of biomass) to natural environmental gradients (i.e. response capacity). We addressed this question with lake phytoplankton, which are at the base of aquatic food-webs, play a major role in their ecosystem processes (e.g. the carbon cycle), and the traits that matter for their environmental responses (e.g. cell size, photosynthetic pigment type and concentration) are well known and can be measured at the individual level [15, 17]. PPCP are found worldwide in surface waters at low concentrations, and hence are classified as micropollutants [1]. They are not completely retained or degraded by waste water treatment plants and are frequently detected in fresh waters in complex mixtures [1, 18, 19]. Their effects on natural systems are poorly understood, but they pose a concern because they are specifically designed to be biologically active [20, 21].

**Fig 1:**
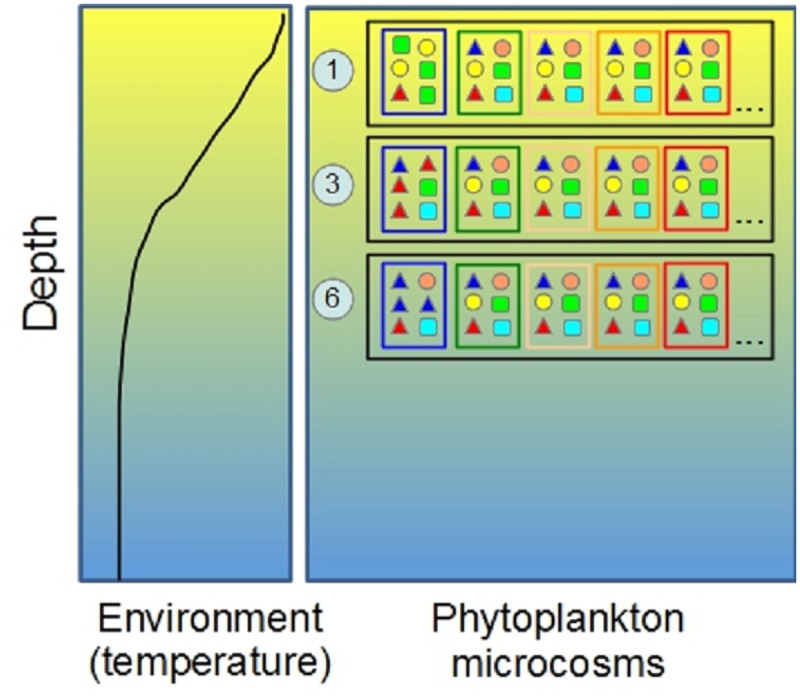
The experimental setup of this study. The temperate eutrophic lake offers a vertical gradient in environmental conditions (black lines in the left plot corresponds to temperature during summer water stratification) and limiting resources (shading in figure, which may correspond to decreasing light / increasing nutrients with increasing depth). Large black boxes represent experimental racks deployed at different depths (1, 3 and 6 m); coloured inner boxes (microcosms) represent different treatments (Figs 2 and 3), and different shapes inside microcosms represent different trait combinations at the start of the experiment.

The objective of this study was to explore how realistic exposure scenarios to PPCP modify the phenotypic structure of natural phytoplankton communities and their productivity in a fluctuating environment. To do so, we performed an *in situ* experiment (Fig 1) in the eutrophic lake Greifensee (Switzerland). For the exposure scenarios, we assembled a mixture of PPCP that mimics the combination of compounds found in European surface waters (Table 1, Table B in S1 Text) [22–24]. We used three different test levels resembling concentrations found in polluted lakes (low exposure), polluted rivers (medium exposure) and waste-water effluents (high exposure) (Table 1, Table B in S1 Text) [18, 25–34]. We tracked changes in community metrics, as well as phytoplankton size and overall phenotypic diversity, using scanning flow-cytometry (SFC). We expected that PPCP would impose a strong selection on phytoplankton community composition [35–38]. Therefore, to understand how PPCP and depth determined temporal changes in phytoplankton community trait composition, we generated expected distributions of phenotypic diversity and productivity from a random assembly of starting communities, and studied the deviation of observed data from null assumptions of community change under the tested scenarios.

**Table 1:** The micropollutant mixture concentrations (ng / L).

Compound	Therapeutic category	Spiked for medium exposure level^$^	Measured for medium exposure level^¶^^	European river concentration (median values)^	Background concentrations in lake water*
**Atenolol**	anti-hypertensive	1000	1065 ± 118	351	7.0 ± 1.4
**Bezafibrate**	lipid regulating	100	97 ± 7	46	0.5 ± 0.3
**Carbamazepine**	anticonvulsant/antidepressant	1000	1205 ± 177	121	50.8 ± 8.9
**Clarithromycin**	Antibacterial	1000	1000^§^	143	NA ^§^
**Diclofenac**	anti-inflammatory	1000	654 ± 151	190	< 5.3~
**Furosemide**	Diuretic	100	46 ± 9	49.5	< 0.8~
**Hydrochlorothiazide**	Diuretic	1000	501 ± 57	174	< 0.9~
**Ibuprofen**	anti-inflammatory	100	132 ± 21	97	12.9 ± 0.9
**Ranitidine**	ulcer healing	10	2.4 ± 0.5	16.5	< 0.9~
**Sulfamethoxazole**	Antibacterial	10	3.7 ± 0.5	7	4.6 ± 1.0
**benzophenone-4**	solar filter	1000	793 ± 106	178	38.9 ± 6.0
**Triclosan**	antibacterial/fungicide	100	68 ± 50	59	< 9~

Initial concentrations for the medium exposure were chosen to resemble median environmental levels in European rivers, rounded up to the nearest order of magnitude (spiked level). Doses spiked in the experimental microcosms were influenced by diffusion/decay of compounds, changing the actual exposure scenarios (measured levels, see also Fig 4 and Fig. H in S1 Text). Background concentrations of micropollutants in lake water during the experiment are also reported.

^¶^ mean ± standard deviation of 3 replicated analyses of inocula at the beginning of the experiment;

^$^spikes for low and high concentrations corresponded to one tenth and ten times the levels reported for medium level, respectively (Tables A-B in S1 Text);

^§^ nominal concentration: due to the low instrumental sensitivity and the non-linear response of the calibration curve, it was not possible to obtain reliable results for clarithromycin in lake water, low and medium dosages;

^ references and information can be found in Methods;

* average among the experimental depths ± standard deviation;

^~^ limit of quantification, calculated in the lake matrix as the concentration giving a signal to noise ratio of 10.

## Material and methods

### Site

Our experimental site was the temperate lowland lake Greifensee, located at 435 m above sea level on the Swiss Plateau, north of the Swiss Alps; it has a maximum depth of 32 m and a surface area of 8.5 km^2^. The lake is surrounded by urban areas and receives micropollutant discharges from wastewater treatment and sewer/storm overflows (Table 1). The experiments were performed near the Eawag automated monitoring station in the lake (47° 21’ 58” N; 8° 39’ 56” E, maximum depth = 20 m) between June 27, 2012 and July 4, 2012. Permission for water monitoring and working in situ was provided by the Office of Waste, Water, Energy and Air of Canton Zürich (http://www.awel.zh.ch/internet/baudirektion/awel/de/service/international.html). No other specific permissions were required since we used PPCP that were already present in the lake (Table 1) and we use them in small amounts within confined microcosms. We confirm that the field studies did not involve endangered or protected species. We collected water samples at all experimental depths to characterise the physico-chemical environment at the start and end of the experiment.

### Experimental procedures

We sampled lake phytoplankton communities from three depths (1, 3 and 6 m), collecting 5L from each using a Niskin bottle, and filtered them through a 100 μm nylon mesh to remove larger zooplankton grazers. Environmental factors such as light, temperature, and nutrients change strongly with depth and frequently over time, thereby altering community composition and trait distributions (Fig 1 and S1 Text, sections 2.3. and 2.4). We mixed subsamples from every depth in equal proportions to create a pooled assemblage, to which we applied the micropollutant treatments at the desired exposure levels, or only vector (controls) (Fig 1). In this way we could examine the effect of a changing habitat (depth) on a mixed community with an assemblage of all phenotypes present in the lake water column, and how the re-organisation of such mixed community interacted with the effects of micropollutants. We kept a subsample from each depth as depth-specific community (formed by reserving 1 L of water from each depth); these communities experienced no manipulation or exposure to PPCP (Fig 1) and acted as a reference for mixed community re-organisation over the 3 experimental depths.

Three depth-reference communities (one for each experimental depth) and four mixed communities (control and 3 micropollutant treatments) were prepared in 4 replicated microcosms of 100 mL for each of the 3 experimental depths (total = 60 microcosms). After spiking with vector (ethanol, for controls and depth-reference communities) or PPCP, 40 mL aliquots from each microcosm were fixed with a filter-sterilised solution of paraformaldehyde and glutaraldehyde (0.01 and 0.1% final concentration, pH 7) and stored at 4°C in the dark for SFC [15, 39]. The remaining 60 mL were transferred into 25 cm long, 2 cm diameter extruded cellulose ester dialysis bags (Spectra/por, Spectrum Europe, Breda, The Netherlands) sealed with nylon clips. The dialysis bags had a molecular cut-off in the range of 100–500 Da that allowed efficient exchange of nutrients, gases, and compounds of similar molecular weight with the outer environment. They were also fully transparent to photosynthetic active radiation (PAR) and highly hydrophilic, a property that strongly reduces biofouling and enables efficient recovery of the inoculated microorganisms [39]. Dialysis bags were randomly placed into PVC tubes (transparent to PAR) mounted into submersible racks (to protect them from damage), and then positioned at 1, 3 and 6 m depths (Fig 1, Fig. A in S1 Text). Depth reference communities were incubated at the same depth that they were sampled at. The total time for experimental preparation was less than 3 h during which the phytoplankton samples were shaded. Given the population growth rates of phytoplankton (generation time ~ 2 days on average in the field), the experiment allowed us to test for trans-generational effects of micropollutants on phytoplankton and the resulting consequences for community structure, trait variation, and functional properties. After one week, all microcosms were transferred to Falcon tubes, fixed as above and stored for SFC.

### Chemicals and exposure levels

PPCP were selected based on their occurrence in the environment [40], as well as on forecasts of their use and sale levels [18, 41]. Experimental concentrations were chosen based on levels measured in the environment, reflecting values observed in European freshwaters [18, 25–34], rounded up to the nearest order of magnitude (Table 1 and Table A in S1 Text). The model mixture of PPCP used here has been previously tested on different organisms showing significant sub-lethal individual effects and interactions, which can be both dose and organism-dependent [22–24]. Experimental levels for each micropollutant were at least one order of magnitude lower than the effective concentrations in standard toxicity tests on algal growth using single species, with the exception of clarithromycin (Table C in S1 Text). Details of the chemicals used and their preparation can be found in S1 Text. We refer to the mixture of pharmaceuticals at experimental concentrations in Table 1 as the “medium exposure”; low and high concentrations correspond to one tenth and ten times the nominal exposure levels reported for medium levels (Table 1, Table C in S1 Text), respectively.

### Flow cytometry

We used the scanning flow-cytometer Cytobuoy (Cytobuoy.com, Woerden, The Netherlands) for analysis, counting and characterisation of phytoplankton [15, 39, 42]. This instrument uses 2 solid-state lasers (488 nm and 635 nm) in a time-resolved mode (scanning) to measure length and pigment fluorescence for all particles between 0.5 to 700 μm in diameter and circa 1 mm in length. The above measures derived by SFC have been shown to respond to variation in environmental conditions, ecological interactions and chemical stress [15, 39]. Relative to analysed volume, phytoplankton concentrations were calculated by treating each hump in the sideward scattering (SWS) signal of each particle as a single cell, to account for colonial taxa [15, 42]; total biomass was determined by summing the wet biomasses of individual particles in each sample [15, 39, 42] (see below).

We sampled each experimental microcosm twice (total Cytobuoy measures per experimental treatment = 8) to get a better resolution on trait diversity and scanned roughly 10,000 particles from each sample. Considering 60 microcosms sampled at two time points (beginning and end of experiment), each analysed by Cytobuoy in two technical repeats, the final database comprised 120 community matrices of scanned phytoplankton particles. We measured total bacterial cell concentrations (used as a covariate in the statistical analyses—see below) in our experimental microcosms using a BD Accuri C6 flow cytometer (S1 Text section 1.4).

#### Estimation of trait values and trait diversity

Raw Cytobuoy data were visually inspected for the distribution of FL signals in order to set gating levels to extract FL particles (phytoplankton) with a length > 1 μm (for an accurate estimation of particle descriptors). We estimated the relative wet phytoplankton biomass (ng) using the total forward scattering (FWS) signal, as reported elsewhere [39, 43], by assuming an ellipsoid shape and that particles had the same density of water (biovolume = wet biomass). We standardised and then used all Cytobuoy particle descriptors (some of which are cross correlated) in a principal component analysis (Table E in S1 Text) to account for all the information on phytoplankton three-dimensional structure, fluorescence properties, cell/colony size and distribution of pigments and other structures within cells [15]. We studied principal components PC1 (accounting for circa 30% of total multivariate trait variance) and PC2 (20% of trait variance) as an aggregated index of phytoplankton morphology: PC1 corresponds approximately to particle pigment packaging and length; PC2 to scattering and shape (Table E in S1 Text). To determine phenotypic diversity, we used the first 37 PCs covering 99% of the trait variance in the data, and the method by Petchey and Gaston [44].

#### Bootstrapping

Our approach to estimate phenotypic diversity required to calculate Euclidean distances among all phytoplankton individual particles across the whole experiment and to build a general dendrogram [45]. To accommodate for computer memory issues, we bootstrapped (without replacement–i.e. jackknife resampling) the whole analysis 100 times by limiting the input dataset to 30,000 randomly extracted particles from the total database at each round of resampling. At each round of the general jackknife resampling, we acquired community structure and functioning metrics and deviations from random assembly standardising the number of individuals sampled per experimental community. This approach required an additional step of bootstrapping, performed within each round of resampling from the general database.

Specifically, at each round of resampling from the general database:

1. For phenotypic diversity calculations, distance matrices and cluster dendrograms were created using the library “*rpud*” for R parallel computing [46], and branch lengths corresponding to individual particles in each microcosm community were extracted from the general cluster dendrogram [45]. Since the calculation of phenotypic diversity is sensitive to the number of individuals present in the sample [44], we estimated this metric from each experimental community using the same number of individuals for all samples, namely the number of individuals present in the smallest of our experimental communities. Phenotypic diversity was therefore bootstrapped 100 times per round of resampling.
2. While calculating phenotypic diversity (100 times per round of resampling), we extracted community functional metrics (particle concentration, cells concentration, total FL of Chl-a, total biomass) and community-wide trait distribution metrics (e.g. mean and variance) for i) a set of focal traits (particle length, number of cells per colony–derived from the SWS signal–and particle biomass–derived from FWS signal), and ii) the first and second principal component of all Cytobuoy descriptors (Table E in S1 Text). The choice of metrics and traits was based on previous studies [15].
3. To study if and how community composition changes were non-random and influenced by our experimental factors, we generated expectations from random re-shuffling of individuals in the communities at the start of the experiment, and compared detected patterns at the end of the experiment with expectations from random assembly. Random expectations were obtained by bootstrapping (jackknife) the starting communities 100 times (per round of resampling from the general database), and by standardising the number of re-sampled particles to the amount of individuals found the communities at the end of the experiment. This was possible since in our experiment the total phytoplankton concentration inside microcosms decreased over the period of study. For all our endpoints of interest, the datum observed for the community at the end of the experiment was ranked in the random distribution of expected values from stochastic assembly, and the normalised rank position saved (Fig. A in S1 Text). In this way we could assess how much treatments and controls deviated from random assembly of starting communities.

At each round of resampling from the general database all the results for steps 1-2-3 were saved, and the final data utilised for plots and further analyses overall represent the mean of 10,000 iterated calculations.

### Analysis of the experiment

The experiment was a full factorial design with depth (3 levels) and micropollutants mixture (4 levels) as factors (replication = 4), plus the reference community at each depth which was not considered here. Data processing, analysis and graphics were performed with the R statistical programming language [46], Matlab (MathWorks, Natick, MA) and SPSS (IBM, Armonk, NY). HPLC-MS/MS data were acquired and elaborated using Analyst Software 1.5 (AB—Sciex, Thornhill, Ontario, Canada).

The factorial design of experiments (DOE) was analysed using a general linear model (GLM) in SPSS and Type III sums of squares. Factors included in the model were depth, micropollutant concentration and their interaction, with initial levels of the response variable and abundance of bacteria (or abundance of phytoplankton for the bacterial model) as covariates. We included bacterial abundance in our statistical model to account for potential effects of heterotrophic bacteria on the persistence of chemicals or recirculation of nutrients in our microcosms, and therefore on community effects. Though depth is technically continuous, the response of biological, physical and chemical variables to depth is highly nonlinear. Assuming a specific shape for the biological response against depth would therefore be imprecise, and so we chose to treat depth, as well as dose, as factors in GLM. Since each experimental bag was sampled twice with the Cytobuoy, we included bag as a random intercept nested within depth, dose and their interaction. Significance, direction of effects and effect size of each factor was estimated from the GLMs. Effect size was estimated as partial Eta-squared (the default effect size measure reported in SPSS). Partial Eta-squared represent the proportion of the total variance in the dependent variable that is associated with the membership of different levels of an independent factor, when other independent factors and interactions are partialled out [47]. This measure of effect size is common and allows the comparison of the magnitude of effects across studies [47].

### Characterisation of chemical stress

The exposure scenarios in this study were confirmed by studying the persistence of chemicals in our membrane-based microcosms by high performance liquid chromatography-tandem mass spectrometry (HPLC-MS/MS), because micropollutants could degrade or diffuse through the dialysis membranes with a rate dependent on their physico-chemical properties. We measured pharmaceuticals and personal care products in our experiments using solid phase extraction and HPLC-MS/MS following the multiresidue analytical method for waste and surface waters previously published previously [48]. For details see S1 Text section 1.3.

In order to characterise phytoplankton exposure to the PPCP mixture, we modelled time trends of chemical concentrations in samples exposed to the high concentration treatment using linear or quadratic models in order to define a function *C*_*(t)*_ describing the change of individual contaminant levels over time (Fig. B in S1 Text). We applied the same compound specific-models obtained for the high concentration treatment to the other exposure levels by translating along the Y-axis to match the measured initial concentrations in the different treatments (Fig. B in S1 Text). This approach was validated by comparing model predictions with observed kinetics from low concentration treatments. Organisms exposed to chemical mixtures are sensitive to mixture composition, concentration and duration of exposure [24, 49]. In order to summarise all this information in a simple way we defined the dosage as follows:

$$dosage=\underset{i}{\sum }\int_{t=0}^{7d}C_{\left(t\right)i}dt$$

where, *C*_*(t)i*_ is a function describing the change of individual contaminant concentrations over time. Being represented by the summed time integrals of the concentrations for all compounds in the mixture, the dosage accounts for the level and time in which each micropollutant *i* contributed to the total mixture exposure. The dosage for lake levels of contamination was calculated in the same way, assuming that the environmental background concentration of micropollutants remained constant during the experimental week.

### Data accessibility

Data and SPSS analysis codes are available from the Dryad Digital Repository at http://dx.doi.org/10.5061/dryad.7rn79.

## Results

### Estimation of factor effects

We first addressed the direct effects of PPCP, depth and their interaction on phytoplankton traits, phenotypic diversity and biomass production of the community at the end of the experiment. Trait distributions were influenced by both PPCP and depth (Table 2). Increasing concentrations of pharmaceuticals significantly reduced variance of all traits without any detectable changes in average trait values (Table 2, and Table H, Fig. C-D in S1 Text). With increasing depth, trait mean values changed (with the exception of average length) and variance generally decreased (Table 2). Interactions between depth and PPCP were non-significant for most individual traits, but influenced variance of PC1 (corresponding to pigment packaging and cell length) and mean of PC2 (corresponding to cell internal structure and shape).

**Table 2:** Effect of micropollutants and depth on individual-level traits and community metrics.

			Factors		
		Metric	Depth	Dose	Interaction
**Trait change**	**PC1 (pigment packaging, size)**	*Average*	**(-) 0.265**	(+) 0.175	(-) 0.231
		*Variance*	**(-) 0.399**	**(-) 0.263**	**(-) 0.357**
	**PC2 (scattering and shape)**	*Average*	**(+) 0.189**	(+) 0.134	**(-) 0.293**
		*Variance*	(-) 0.072	**(-) 0.233**	(+) 0.148
	**Length (μm)**	*Average*	(+) 0.072	(+) 0.092	(-) 0.227
		*Variance*	**(-) 0.315**	**(-) 0.224**	(-) 0.281
	**Biomass (ng)**	*Average*	**(-) 0.204**	(+) 0.100	(-) 0.195
		*Variance*	**(-) 0.265**	**(-) 0.253**	(+) 0.109
**Community effect**	**Phenotypic diversity**	*Total*	**(-) 0.403**	**(-) 0.427**	**(-) 0.282**
	**Cells mL**^**-1**^	*Total*	**(+) 0.454**	**(+) 0.724**	**(-) 0.316**
	**Biomass (μg L**^**-1**^**)**	*Total*	**(+) 0.284**	**(+) 0.677**	(-) 0.186
	**Chl-a fluorescence**	*Total*	**(+) 0.156**	**(+) 0.300**	**(-) 0.409**

The strength of the effect is reported in terms of partial Eta-squared (proportion of the total variance explained by each factor in the GLM). The direction of effects (from the parameter estimate of the GLM) is reported in parentheses (positive / negative). PC1 and PC2 represent the first and second principal components of all measured traits, respectively. Statistically significant effects (p < 0.05) are highlighted in bold. For details of trait loadings on PCs and model details, including random effects, see Tables E and H in S1 Text.

As a consequence of reduction in trait variance, also phenotypic diversity was reduced by both depth and PPCP, with a significant negative (antagonistic) interaction (Table 2, Table H, Fig. D-E in S1 Text). Phytoplankton community cell concentrations, biomass and Chl-a FL significantly increased with both depth and PPCP, with pharmaceuticals having a stronger effect. Significant effect sizes of PPCP on traits and community properties were very large, with values of estimated partial Eta-squared being always larger than 0.2 [47] and reaching sizes as large as 0.724 in the case of cell densities and 0.677 for community total biomass (Table 2). Interactions between depth and PPCP were negative and significant also in the case of Chl-a FL and cell concentrations. Increases in biomass were primarily driven by higher phytoplankton cell numbers rather than changes in cell length (Table 2, Table H, Fig. C-E in S1 Text).

### Temporal responses of experimental communities

During the experiment, lake Greifensee was stratified and the thermocline was at circa 7 m (Fig. F in S1 Text). The three lake depths sampled and utilised for the experiment represented different habitats with regards to chemistry and physics, with 1 and 3 m being more similar to each other than 6 m depth, which was the coldest and poorer environment in terms of light and P-PO4 (Fig. F in S1 Text). Environmental conditions also slightly changed during the seven days of the experiment since the weather became cloudy and cold, with moderate rain events (Fig F in S1 Text). Since rain events were not strong enough to bring in nutrients from the catchment or de-stabilise the water column, the lake environment became colder, darker and slightly more oligotrophic at the end of the experimental period.

**Fig 2:**
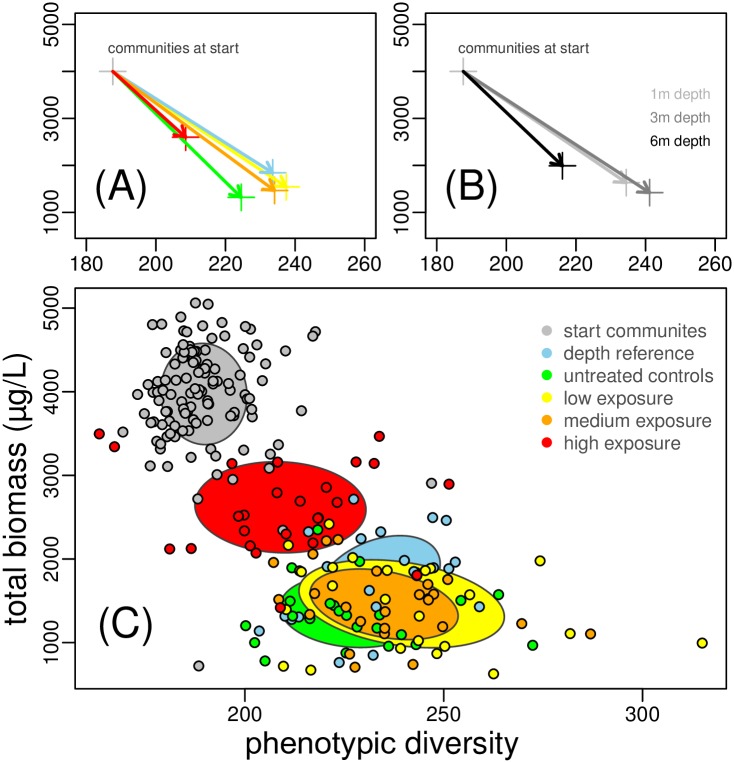
Effects of increasing micropollutant exposure (A-C) and depth (B) on phytoplankton phenotypic diversity and total biomass in experimental communities at the beginning and at the end of the experiment. A-B) arrows connect centroids of each set of treated communities highlighting temporal changes (axes labels as in C); C) ellipses denote 95% confidence intervals for each set of treated communities.

All experimental communities experienced an increase in phenotypic diversity and a decrease in total biomass (Fig 2). Microcosms exposed to the high PPCP treatment and those deployed at the highest depth (6 m) remained more similar to the starting communities in terms of phenotypic diversity and total biomass, with PPCP having a stronger effect than depth (Fig 2A and 2B). The highest PPCP exposure level showed the most significant deviations in phytoplankton phenotypic diversity and biomass when compared to untreated controls (p < 0.001), while low and medium treatments were mostly marginally insignificant (p < 0.1) (Table 3). Phytoplankton communities spiked with the highest PPCP levels remained clearly separated from all the other treatments with regards to their phenotypic structure and function (total biomass), in between starting communities and the cluster formed by controls and the other exposure levels (Fig 2C).

**Table 3:** Statistical differences between phytoplankton communities spiked with micropollutants and untreated controls in terms of trait diversity and total biomass.

Exposure	Phenotypic diversity			Total biomass (μg L^-1^)		
	Mean Difference	Std. Error	p-value	Mean Difference	Std. Error	p-value
**low**	-12.8	4.28	0.004	-86.3	50.66	0.095
**medium**	-7.6	4.29	0.082	-92.3	52.14	0.083
**high**	16.9	4.46	0.000	-1249.4	51.37	0.000

The table reports general linear model-estimated marginal means, standard errors, and *F*-test probability values for comparisons between untreated controls vs low, medium and high exposure levels (as factors).

### Mechanisms of community change

**Fig 3:**
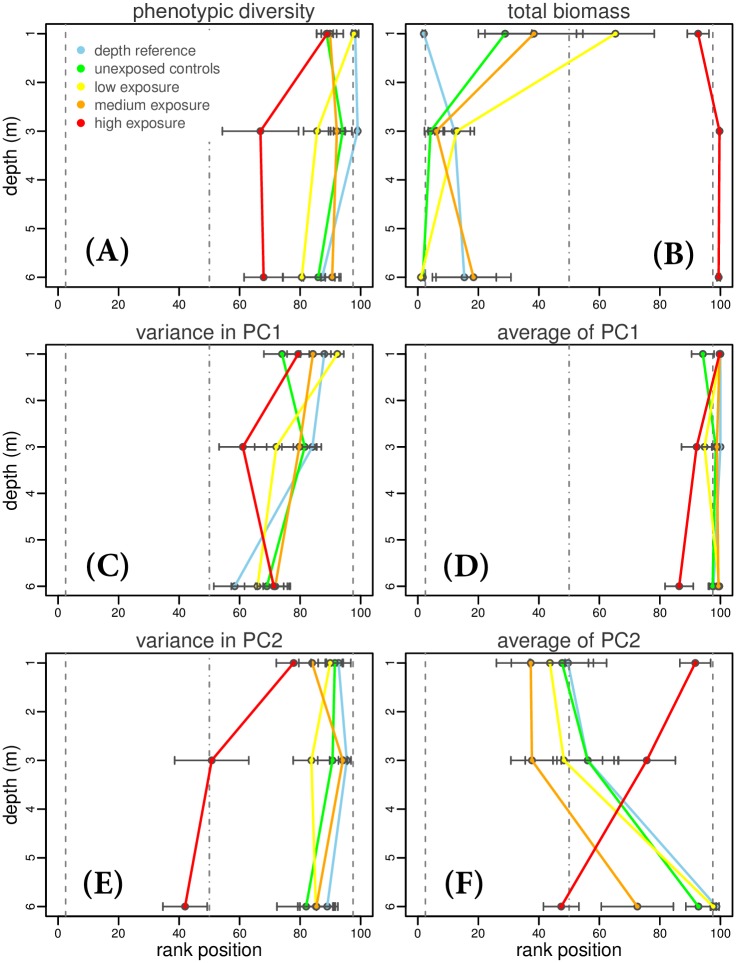
Deviation of microcosms at the end of the experiment from a null expectation created by random assembly of starting communities, as a function of depth. Median, 2.5% quantile and 97.5% quantile are represented by dashed lines. Observations at the extremes of the distribution (< 2.5% or > 97.5%) constitute strong evidence for selection on community structure and function: if rank positions (X-axis) of the final community deviate towards either extreme, depth (Y-axis) or micropollutants (colour coding) influenced phenotypes and their abundance. Note that low and high normalised rank positions are indicative of small and large values of the actual variable, respectively. Points represent mean ± standard error.

During the course of the experiment, both depth and PPCP imposed selection on community phenotypic structure and biomass (Fig 3). Most experimental microcosms differed strongly from expectations of random assembly of phenotypes, particularly unexposed communities (blue and green lines, Fig 3). The depth reference communities represented in our experiment the control for natural assembly patterns of phenotypes, since they were unbiased by the initial mixing and were not spiked with micropollutants. Unexposed controls mostly converged to the same phenotypic structure of the depth reference communities during the experimental period (Fig 3A and 3C–3F). Low and medium PPCP treatments showed similar structures to background controls, with slight deviations (Fig 3, and Fig G in S1 Text). In the high PPCP treatment, however, phytoplankton communities remained clearly more similar to a random assembly of starting phenotypes compared to depth references and the other treatments (Fig 3A, 3C and 3E, Fig. G in S1 Text). Also experiments at 3 and 6 m depths showed to be structurally more similar to a random assembly of starting communities. Similar to what was observed in Fig 2, the impact of high levels of PPCP on the direction of community change was much stronger than the depth gradient, and during the experiment PPCP induced a significant deviation in total biomass and trait mean values compared to expectations from the other treatments or a random reshuffling of starting communities (Fig 3B, 3D and 3F, Fig. G in S1 Text).

### Characterisation of chemicals stress

PPCP in the experimental mixture showed different kinetics. There were two groups of compounds: stable (including: sulphamethoxazole, diclofenac, benzophenone-4, bezafibrate and furosemide) and with decay (including: ibuprofen, atenolol, hydrochlorothiazide, carbamazepine, ranitidine, triclosan and clarithromycin) (Table A, F, Fig. B in S1 Text). The compounds with no net declining trend were those that had and initial concentration which was closer to the lake background levels. Concentrations and trends were not significantly different at different depths (p < 0.05, Kruskal-Wallis test) with the exception of clarithomycin decay at 1 m and 3 m depths (p = 0.04), and slight difference (p = 0.06) for furosemide at 1 m and 6 m depths (Table G in S1 Text). The depth factor, therefore, did not have a general influence on kinetics of our compounds.

**Fig 4:**
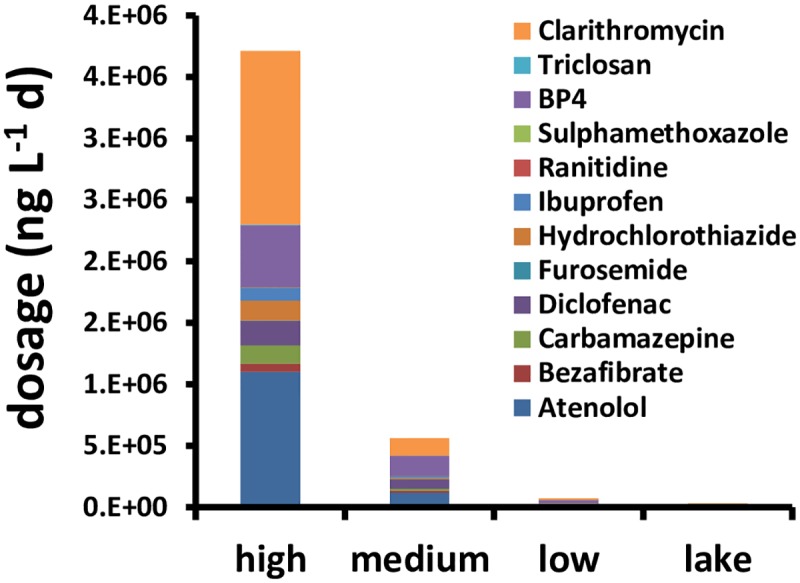
Characterisation of dosages, calculated as the time integral of the concentrations of chemicals composing the mixture. It summarises the information of mixture composition, total level and duration of exposure in the lake and in the experimental microcosms.

Treatment with different initial spikes generated different exposure scenarios in terms of duration and composition of the mixture (Fig 4, and H in S1 Text). All chemicals in the low concentration treatments (with the exception of clarithromycin and atenolol) converged to background levels of lake pollutants in about two days (Fig. B and H in S1 Text). Similarly most of compounds in medium concentration samples converged to background levels of pollutants after about 5 days. At 7 days low and medium concentrations were close to background levels, with the exception of clarithromycin and atenolol that remained rather stable at their initial levels over time, while high concentration treatment was still above background levels for all pollutants in the mixture (Fig 4).

## Discussion

Previous work has shown how PPCP and their mixtures can affect natural algal communities in environmentally relevant scenarios [50, 51]. Exposure to drug mixture has been reported to influence both species composition and growth of algal assemblages [35–38, 52]. In particular, a trait-based analysis of phytoplankton communities exposed to triclosan in mesocosm enclosures has revealed that this disinfectant can induce cascading effects, from individual responses to population and community changes [39]. Our results show that high micropollutant levels, comparable to PPCP concentrations found downstream of waste-water effluents, imposed significant effects on the phenotypic composition of natural phytoplankton communities. To our knowledge, the present study is the first report of PPCP mixtures being able to reduce individual-level trait diversity in ecological communities, and that pollution-induced phenotypic changes interfere with community assembly over natural environmental gradients.

Experimental microcosms exposed to micropollutants exhibited a general decrease in trait variance relative to controls, and communities were not able to converge to similar patterns of phenotypic diversity and biomass of the reference and control communities (Figs 2 and 3). This suggests that PPCP modified the fundamental relationship between phytoplankton community structure (phenotypic profile) and biomass production, similarly to what has been recently observed for the herbicide atrazine [53], a very specific chemical stressor affecting photosynthesis. Reduction of trait variance in the phytoplankton communities suggests that micropollutants acted as a strong environmental filter [54]. In theory, PPCP could also have influenced community structure by modifying the strength of species interactions [11, 53, 55]. In our data, however, we did not observe evidence for changes in trait distributions (e.g. even spacing of traits) associated with variation in the strength of competition (see S1 Text, section 2.4). This indicated that community assembly over the lake depth gradient in habitats was driven by environmental filtering processes and that exposure to PPCP shifted natural filters influencing the structure and function of assemblages.

It has been hypothesised that, when faced with multiple co-occurring environmental changes, the ability of natural communities to adapt to fluctuating conditions may depend on phenotypic diversity [7, 56] and the trade-offs between traits that determine tolerance to different stressors [57, 58]. In our study, three likely environmental drivers for the temporal change during the experiment were the exclusion of large grazers and a decrease in lake temperature and available resources (Fig. C in S1 Text). Control experimental communities experienced an increase in phenotypic diversity and a decrease in cell densities and total biomass over the period of study (Fig 2). The net effect of high exposure to PPCP was a reduction in phenotypic diversity and a hindering of biomass loss. The negative interaction between micropollutants and the environmental gradients offered by depth and temporally changing conditions (Table 2 and Fig 3) suggests a fundamental trade-off between tolerance to PPCP, which may select for specific trait combinations (i.e. lower phenotypic diversity, higher biomass—Figs 2 and 3 and G in S1 Text), and the capacity of a community to respond to fluctuating environmental challenges.

While higher cell densities and biomass at greater depths can be explained by a subsurface primary production maximum of summer phytoplankton communities (S1 Text), we can only speculate about the mechanisms that led to greater amounts of total biomass in communities exposed to high levels of PPCP. Total biomass of a community can vary due to ecological mechanisms (the species composition of communities), physiological responses (e.g. changes in the size of individual organisms), or evolutionary processes (changes in the size structure of populations due to turnover of different genotypes) [14]. Very few studies have been able to tease apart the relative importance of these processes in community biomass changes due to chemical pollutants, and evidence suggests that all these processes can be important, especially in phytoplankton [14, 39, 53]. Our experiment was not designed to study the above eco-evolutionary mechanisms.

In our data, higher cell densities in the high micropollutant exposure may have been due to an indirect ecological effect of heterotrophic bacterial activity maintaining a high local nutrient recirculation and availability for phytoplankton growth. Heterotrophic bacterial abundance, although positively influenced by PPCP levels, decreased in our microcosms over the experimental period and had no statistically significant effect on phytoplankton response variables (Table H in S1 Text). Alternatively, micro-zooplankton might have been present in our microcosms and therefore influenced patterns in biomass (Fig 2) by, for example, having stronger grazing effects in controls and low exposure treatments (assuming that such organisms would have been inhibited by high PPCP levels). This is however unlikely since micro-zooplankton grazers are known to be size selective feeders and would have reduced both the biomass as well as the mean particle length and the overall phenotypic diversity in grazed communities [13, 59], which was not evident in our data (Table 2, Figs 2 and 3). An alternative intriguing hypothesis to explain the low phenotypic diversity, higher cell densities and biomass observed in communities exposed to high levels of PPCP is a potential selection of the pollutants mixture for a certain phytoplankton group. A recent meta-analysis suggests that chemicals, including PPCP, could favour cyanobacteria by suppressing the growth of competing algae, and/or by direct stimulation cyanobacterial growth [60].

Physiological stimulation of growth has been shown to occur in populations and communities exposed to sub-lethal levels of PPCP [24, 39, 51]. However, previous studies focusing on the mixture of PPCP used here showed that, in vitro, PPCP can induce generic stress responses in eukaryotic cells, inhibiting primary metabolism and inducing cell-cycle arrest (not senescence or apoptosis) at high doses [22, 23]. The observed maintenance of greater cell concentrations and biomass in the highest PPCP treatments could be explained by induction of algal stress response mechanisms and inhibition of cell-cycle progression, which would slow down generation time, population growth, and community turnover in species (i.e. less response capacity). The fact that we did not detect effects of PPCP on mean length, biomass or number of cells per colony of individual phytoplankton particles (Table H in S1 Text), which was observed in previous studies [39], supports the hypothesis of a slowing down of cell and population responses. This effect could have been mediated by the antibiotic clarithromycin, which was present in the highest exposure scenario at concentrations that were two orders of magnitude above the generalised environmental background (Table 1, Fig 4 and Table B, Figure H in S1 Text). This compound is known to be very toxic to algae (Table C in S1 Text) and able to interact with other micropollutants [61]. On the other hand, the small effects observed for the medium and low micropollutants dosages suggests that natural populations in Lake Greifensee, which is subjected to seasonal and inter-annual cycles of PPCP [62, 63], may be adapted to fluctuations of pollutants at levels that are similar to or slightly above background contamination (Fig 4 and Fig. H in S1 Text).

We report important response patterns of natural phytoplankton communities to diffuse and common water-borne pollutants. Our results contribute to the understanding of environmental effects of chemicals by suggesting a fundamental trade-off between the ability of natural communities to maintain phenotypic diversity under sub-lethal stress from widespread chemical pollutants, and their capacity to re-organise structure and function when facing additional environmental challenges. We recognised that filling in the knowledge gap of environmental effects of chemical pollutants on ecological systems is a daunting challenge, particularly when sub-lethal levels of chemicals are interacting with other environmental stressors [49, 58]. In this context, our work suggests that future research should more systematically target the effects that pollutants have on the mechanisms that maintain biodiversity and functioning over environmental gradients in natural ecological systems, including experiments and theoretical studies of how individual-level responses to multiple stressors influence biodiversity and scale through population and community interactions to affect ecosystem functions. Such information might be crucial for management and policy to protect ecosystem processes in human impacted environments.

## Supporting information

S1 Text This file contains SI methods, results, discussion and references. Specific results include Tables A-H and Figures A-H.
(DOCX)
