# Supplementary material for: Water-borne pharmaceuticals reduce phenotypic diversity and response capacity of natural phytoplankton communities
Source: PLoS One. 2017 Mar 22;12(3):e0174207. doi: 10.1371/journal.pone.0174207 (PMC5362198; doi:10.1371/journal.pone.0174207)
Supplement: S1 Text — Specific results include Tables A-H and Figures A-H. (DOCX) [file pone.0174207.s001.docx]

# Supporting Information Text for

**Water-borne pharmaceuticals reduce phenotypic diversity and response capacity of natural phytoplankton communities**

F Pomati, J Jokela, S Castiglioni, M K Thomas, L Nizzetto

**This PDF file includes:**

SI Methods

SI Results and discussion

SI Tables - Tables A to H

SI Figures - Figures A to H

SI References – 1-24**SI methods**

- 1. *Chemicals and dosage*

The reference standards were purchased from Sigma-Aldrich (Sigma-Aldrich Co., Dorset, UK), ICN Biochemicals (Meckenheim, Germany), and GlaxoSmithKline (Philadelphia, PA). The stock solution of the experimental mixture was prepared by combining single drugs in ethanol to reach a dose 20,000 times higher than the experimental concentration reported in Table C, and stored at -20°C. The stock solution was then diluted in experimental microcosms to reach the desired exposure level. From here on, we refer to the mixture of pharmaceuticals reported in Table C as the ‘medium’ concentration; low and high concentrations correspond to one tenth and ten times the nominal exposure levels reported for medium, respectively.

1.2 *Micropollutants persistence*

In parallel with the main experiment, we studied the persistence of the micropollutants in our experimental conditions within the membrane-based microcosms. The 7 starting communities (see Methods in main text and Fig. A) were mixed and three 50 mL samples were taken from each for chemical analysis of initial micropollutants levels. Samples were stored on ice in the dark for a few hours until extraction. We also prepared an additional set of microcosms (45 in total) using the low and high treatment communities. Our selected micropollutants have molar masses ranging between 250 and 320 g mol^-1^, and they could diffuse through the dialysis bags and equilibrate with the lake background levels of pollution. We deployed four sets each of triplicate bags containing 50 mL of the high and low micropollutants dosed communities at each experimental depth as in the main experiment. These sets of bags were sacrificed after 6, 24, 48 and 168 h (7 days), respectively, stored on ice and extracted for chemical analysis.

- 1. *Analytical chemistry*

We measured pharmaceuticals and personal care products in our experiments using solid phase extraction (SPE) and high performance liquid chromatography-tandem mass spectrometry (HPLC-MS/MS), following the multiresidue analytical method for waste and surface waters previously published [1], with slight modifications. Samples (50 mL) were filtered through a Whatman GF/F membrane, acidified to pH 2.0 with 37% HCl, spiked with labelled internal standards and solid-phase extracted using mixed reverse-phase cation exchange cartridges (Oasis-MCX, Waters Corp., Milford, MA). Cartridges were then vacuum-dried for 10 min and eluted with 2 mL of methanol and 2 mL of a 2% ammonia solution in methanol. The eluates were pooled, dried under a nitrogen stream, re-dissolved in 200 µl MilliQ water and transferred into glass vials for instrumental analysis.

The analysis was performed using a two Series 200 pumps and a Series 200 auto sampler (Perkin-Elmer, Norwalk, CT) HPLC system and an API 3000 triple quadrupole mass spectrometer, equipped with a turbo ion spray source (AB - Sciex, Thornhill, Ontario, Canada) MS system. Compounds were quantified by selected reaction monitoring using both the positive and negative ionisation modes. A 10-min gradient was used for chromatographic separation using formic acid 0.1% in water for analysis in positive mode and triethylamine 0.05% for analysis in negative mode as solvents A (gradient from 98% to 2% in 10 min), and acetonitrile as solvent B. The flow rate was 200 µL/min and the injection volume were: 20 µL (positive mode) and 10 µL (negative). Quantification was performed by isotope dilution using the two highest most abundant precursor/product ion transitions. Retention times were also compared with reference standards to identify the compounds. The analytical method was fully validated following the guidelines of the European Decision 2002/657/EC39 [2] in order to ensure high quality and repeatability of results. The analytical conditions used for analyses and quantification of the selected analytes are reported in Table D. Concentrations of the studied chemicals obtained during the experiment (0 - 7 days) are reported in Table A.

- 1. *Flow-cytometry settings*

Length of particles was measured from light scattered (908 nm) by particles passing through the instrument, which is measured at two angles, forward (FWS) and sideward (SWS), providing information on length and shape of particles. The fluorescence (FL) emitted by photosynthetic pigments in algal cells is detected at three different wavelengths: red (FL.Red and FL.2.Red from the 488 and 635 nm lasers, respectively), orange (FL.Orange) and yellow (FL.Yellow) signals were collected in ranges of 668 – 734 (chlorophyll-a), 601–668 (phycocyanin) and 536– 601 nm (phycoerythrin and carotenoids), respectively. Together, this generated 54 descriptors of 3D structure and fluorescence of each particle. Further details about the instrument design and settings can be found elsewhere [3, 4]. Digital data acquisition was triggered by the sideward scatter (SWS) signal with a trigger-level of 100 mV. Laser alignment (using FL and non-FL beads of 1 and 4 μm) and pump calibration were performed prior to sample analyses. Each microcosm was sampled and analysed twice (2 repeated measures with identical instrument settings).

We measured total bacterial cell concentrations (used as a covariate in the statistical analyses - see below) in our experimental microcosms using a BD Accuri C6 flow cytometer equipped with a 488 nm solid-state laser, following dilution of samples and the addition of SYBR Green I (Life Technologies Ltd., Paisley, UK) as reported elsewhere [5].

- 1. *Analysis of physical and chemical data*

We analysed physical and chemical variables [6] to understand how the water environment changed over the course of the experiment and to test if micropollutants concentrations were affected by the lake chemistry and physics at different depths. Limnological data comprised temperature, conductivity, pH, alkalinity, N-NO3 and P-PO4, measured at 1, 3, and 6 m depths at the beginning and at the end of the experiment. Water variables were utilized to study differences among lake depths by principal component (PCA) and cluster analyses. The trends of different compounds at each individual depth were explored and compared by PCA built on the matrix of correlation between standardised concentration data. Spearman non-parametric test was used to test the null hypothesis that different compounds had the same behaviour. In order to assess if depth had a significant effect on individual compound concentration trends we used the non-parametric Kruskal-Wallis test.

1. **SI Results and Discussion**
   1. *Characterisation of environmental gradients*

The experiment was performed during one week between the end of June and early July 2012. Over the entire summer (June-September), lake Greifensee is stratified and the thermocline is generally between 6 and 8 m (circa 7 m during our experiment), with the peak of primary production at circa 5 m depth (data not shown). When the lake is stratified and stable, phosphorus (the limiting factor for phytoplankton growth in lakes) is generally locked into algal biomass and P-PO4 (the free available form of P) is depleted in the photic zone (often below our detection limits of 1 µg/L), while waters deeper than 15 m are extremely rich in this essential nutrient (up to circa 100 µg/L at the bottom).

Environmental conditions changed during the temporal framework of the experiment (7 days). After the onset of the experiment the weather was sunny and stable for 4 days, with a steady increase in temperature (Fig F, panel A). On the 1^st^ of July the weather changed to cold and cloudy, with moderate rain events (Fig F, panels A-B). The lake environment became colder, darker (the weather was cloudy) and more oligotrophic at the end of the experimental period (Fig. F, panels C-G). The three lake depths sampled and utilised for the experiment represented different habitats with regards to chemistry and physics both at the beginning and at the end of the experiment (Fig. F, panels C-H).

- 1. *Characterisation of phytoplankton community change*

The three lake depths sampled and utilised for the experiment were characterised by different phytoplankton communities in terms of diversity, trait distributions and biomass (see dark grey dots in Figs C-D-E). The interacting gradients of light and available nutrients in deep lakes generally determine deep Chl-a and productivity maxima in summer [7, 8]. As a consequence of exclusion of zooplankton from dialysis bags and the changing environmental conditions (Fig. F), the experiment captured a transition period in which phytoplankton communities at all depths decreased in their overall abundance and became more diverse (Fig. 2, main text).

Individual algal particles became slightly larger over time (Fig. C, panel E, note however that differences between controls and treatments in individual-level biomass are not significant), but less abundant (Fig. E, panels B and D). The reduction in total phytoplankton community biomass was likely due to colder and cloudy weather, with no detected nutrient inputs from strong rain-driven runoff or wind driven upwelling, that reduced overall algal growth and likely determined turnover in species composition, with the dominant group / species at the beginning of the experiment (warm and sunny weather) decaying in biomass when environmental conditions changed. Such rapid day-to-day shifts in lake phytoplankton abundance due to weather changes are common and have been previously described [3, 9]. Decay in abundance of a dominant phytoplankton group as a consequence to fluctuating environmental conditions would also free other organisms from competitive constraints [10] and explain the general increase in community-wide trait variance and shifts of mean trait values that we have observed in our experiment (Fig. 3 and Fig. G). These represent clear signatures of changing environmental filters: we did not observe significant changes in the even spacing of traits (standard deviation of nearest neighbour distance along the trait axis divided by the range – SDNDr, data not shown), suggesting that community assembly was not significantly influenced by species interactions during the experiment [11, 12]. Additionally, an increase in phenotypic diversity was also expected as a response to exclusion of large generalist zooplankton (Daphnids are the most dominant grazers in the studied period), whose grazing reduces overall phenotypic diversity [12].

For the estimation of phenotypic diversity, we used Euclidean distances and average linkage to create distance matrices and dendrograms, respectively [13, 14], but our results were robust with regards to the choice of distance measure and linkage method (data not shown). With this approach, phenotypic diversity is not mathematically constrained to be directly positively correlated with species richness and thus allows unbiased tests of selection on standing phenotypic diversity [13].

- 1. *Characterisation of chemicals stress*

As expected from previous work [15-17], chemical compounds in the experimental mixtures showed different kinetics. There were two groups of compounds: stable (including: sulphamethoxazole, diclofenac, benzophenone-4 (BP4), bezafibrate and furosemide) and with decay (including: ibuprofen, atenolol, hydrochlorothiazide, carbamazepine, ranitidine, triclosan and clarithromycin) (Tables A and F, Fig. B).

The compounds with no net declining trend were those that had and initial concentration which was closer to the lake background levels. Concentrations and trends were not significantly different at different depths (p<0.05, Kruskal-Wallis test) with the exception of clarithomycin decay at 1 m and 3 m depths (p = 0.04), and slight difference (p = 0.06) for furosemide at 1 m and 6 m depths (Table F). The depth factor, therefore, did not have a general influence on kinetics of our compounds. The rate of degradation is expected to be sensitive to the level of solar radiation which is a strong inverse function of depth. Although photodegradation might have occurred in the bags, the lack of differences in trends along depth suggests that compound decay in the bags was not controlled by photodegradation, but was likely associated to the rate of diffusion from the bags or interaction with the bag surface. These are expected to be a function of temperature and pH (and therefore depth), however the temperature gradient observed during the course of the experiment (with a maximum range of 6°C measured during stable water column conditions at the beginning of the experiment) appeared to be insufficient to generate significant differences.

**SI References**

1. Castiglioni S, Bagnati R, Calamari D, Fanelli R, Zuccato E. A multiresidue analytical method using solid-phase extraction and high-pressure liquid chromatography tandem mass spectrometry to measure pharmaceuticals of different therapeutic classes in urban wastewaters. J Chromatogr A. 2005;1092(2):206-15. Epub 2005/10/04. doi: S0021-9673(05)01428-7.

2. Organization UNID. Complying with ISO 17025. A practical guidebook for meeting the requirements of laboratory accreditation schemes based on ISO 17025: 2005 or equivalent national standards:. Vienna2006.

3. Pomati F, Jokela J, Simona M, Veronesi M, Ibelings BW. An Automated Platform for Phytoplankton Ecology and Aquatic Ecosystem Monitoring. Environmental science & technology. 2011;45:9658-65. doi: 10.1021/es201934n. PubMed PMID: 21981777.

4. Fontana S, Jokela J, Pomati F. Opportunities and challenges in deriving phytoplankton diversity measures from individual trait-based data obtained by scanning flow-cytometry. Frontiers in Microbiology. 2014;5:324-. doi: 10.3389/fmicb.2014.00324.

5. Hammes F, Broger T, Weilenmann HU, Vital M, Helbing J, Bosshart U, et al. Development and laboratory-scale testing of a fully automated online flow cytometer for drinking water analysis. Cytometry Part A. 2012;81 A(10):508-16. doi: 10.1002/cyto.a.22048.

6. Zhang L, Thygesen UH, Knudsen K, Andersen KH. Trait diversity promotes stability of community dynamics. Theoretical Ecology. 2012;6(1):57-69. doi: 10.1007/s12080-012-0160-6.

7. Reynolds CS. Ecology of Phytoplankton. Cambridge: Cambridge University Press; 2006.

8. Litchman E, Klausmeier Ca. Trait-Based Community Ecology of Phytoplankton. Annual Review of Ecology, Evolution, and Systematics. 2008;39(1):615-39. doi: 10.1146/annurev.ecolsys.39.110707.173549.

9. Jennings E, Jones S, Arvola L, Staehr Pa, Gaiser E, Jones ID, et al. Effects of weather-related episodic events in lakes: an analysis based on high-frequency data. Freshwater Biology. 2012;57(3):589-601. doi: 10.1111/j.1365-2427.2011.02729.x.

10. Dugenne M, Thyssen M, Nerini D, Mante C, Poggiale J-C, Garcia N, et al. Consequence of a sudden wind event on the dynamics of a coastal phytoplankton community: an insight into specific population growth rates using a single cell high frequency approach. Frontiers in Microbiology. 2014;5(September):1-14. doi: 10.3389/fmicb.2014.00485.

11. Kraft NJB, Ackerly DD. Functional trait and phylogenetic tests of community assembly across spatial scales in an Amazonian forest. Ecological Monographs. 2010;80(3):401-22. doi: 10.1890/09-1672.1.

12. Pomati F, Kraft NJB, Posch T, Eugster B, Jokela J, Ibelings BW. Individual cell based traits obtained by scanning flow-cytometry show selection by biotic and abiotic environmental factors during a phytoplankton spring bloom. PloS one. 2013;8(8):e71677-e. doi: 10.1371/journal.pone.0071677.

13. Petchey OL, Gaston KJ. Functional diversity (FD), species richness and community composition. Ecology Letters. 2002;5(3):402-11. doi: 10.1046/j.1461-0248.2002.00339.x.

14. Petchey OL, Gaston KJ. Functional diversity: back to basics and looking forward. Ecology letters. 2006;9(6):741-58. doi: 10.1111/j.1461-0248.2006.00924.x.

15. Zuccato E, Castiglioni S, Fanelli R. Identification of the pharmaceuticals for human use contaminating the Italian aquatic environment. Journal of hazardous materials. 2005;122(3):205-9. doi: 10.1016/j.jhazmat.2005.03.001.

16. Castiglioni S, Bagnati R, Fanelli R, Pomati F, Calamari D, Zuccato E. Removal of pharmaceuticals in sewage treatment plants in Italy. Environmental Science and Technology. 2006;40(1):357-63.

17. Loos R, Carvalho R, António DC, Comero S, Locoro G, Tavazzi S, et al. EU-wide monitoring survey on emerging polar organic contaminants in wastewater treatment plant effluents. Water research. 2013;47(17):6475-87. doi: 10.1016/j.watres.2013.08.024.

18. Yamashita N, Yasojima M, Nakada N, Miyajima K, Komori K, Suzuki Y, et al. Effects of antibacterial agents, levofloxacin and clarithromycin, on aquatic organisms. Water Science and Technology. 2006;53:65-72. doi: 10.2166/wst.2006.338.

19. Isidori M, Nardelli A, Parrella A, Pascarella L, Previtera L. A multispecies study to assess the toxic and genotoxic effect of pharmaceuticals: Furosemide and its photoproduct. Chemosphere. 2006;63:785-93. doi: 10.1016/j.chemosphere.2005.07.078.

20. Cleuvers M. Mixture toxicity of the anti-inflammatory drugs diclofenac, ibuprofen, naproxen, and acetylsalicylic acid. Ecotoxicology and environmental safety. 2004;59(3):309-15. doi: 10.1016/S0147-6513(03)00141-6.

**Table A.** Concentration (ng / L, mean of 3 replicates ± standard deviation) of chemicals in experimental inocula at the beginning of the experiment and within selected bags during the course of the experiment. Na= not analysed. * Limit of quantification, calculated in the lake matrix as the concentration giving a signal to noise ratio of 10.

| **Sample** | **Atenolol** | **Bezafibrate** | **Carbamazepine** | **Diclofenac** | **Furosemide** | **Hydrochlor.** | **Ibuprofen** | **Ranitidine** | **Sulphamet.** | **Clarithromycin** | **Triclosan** | **BP4** |
| --- | --- | --- | --- | --- | --- | --- | --- | --- | --- | --- | --- | --- |
| **D1 T0** | 109±2 | 11±0.5 | 159±5 | 85±13 | 6.6±0.9 | 64±5 | 20±2 | 1.2±0.2 | 3.8±0.9 | na | 12±4 | 132±14 |
| **D2 T0** | 1065±118 | 97±7 | 1205±177 | 654±151 | 46±9 | 501±57 | 132±21 | 2.4±0.5 | 3.7±0.5 | na | 68±50 | 793±106 |
| **D3 T0** | 9595±423 | 403±31 | 6899±204 | 2428±17 | 195±49 | 2199±223 | 1031±72 | 31±16 | 6.2±3.3 | 10914±1432 | 569±73 | 2755±306 |
| **D3 6h 1m** | 8573±367 | 344±33 | 3270±24 | 1636±180 | 44±4 | 726±58 | 947±65 | 21±4 | 5.4±2.7 | 10300±676 | 42.7±16 | 2606±225 |
| **D3 6h 3m** | 8398±370 | 343±42 | 3398±76 | 1246±333 | 27±4 | 590±82 | 905±49 | 20±5 | 6.2±2.5 | 8382±237 | 41±27 | 2542±464 |
| **D3 6h 6m** | 8645±337 | 285±20 | 3626±286 | 889±190 | 14±0.5 | 477±50 | 863±22 | 18±5 | 5.3±0.7 | 8129±1310 | 58±0.7 | 2226±301 |
| **D3 24h 1m** | 7803±560 | 271±38 | 1390±212 | 1164±247 | 36±2 | 202±32 | 868±77 | 20±5 | 5.3±1.9 | 9753±2586 | 17±8 | 2200±175 |
| **D3 24h 3m** | 7247±382 | 268±32 | 1278±78 | 888±92 | 21±2 | 172±14 | 784±35 | 16±4 | 5.3±1.0 | 8698±1075 | 18±4 | 2103±193 |
| **D3 24h 6m** | 7525±60 | 275±22 | 1556±181 | 720±181 | 14±3 | 196±26 | 821±53 | 19±1 | 5.5±0.9 | 9246±968 | 14±5 | 2217±401 |
| **D3 48h 1m** | 7489±954 | 334±56 | 961±381 | 1457±301 | 30±13 | 54±20 | 921±198 | 17±4 | 7.2±3.2 | 9861±106 | 6.8±1.4 | 2407±447 |
| **D3 48h 3m** | 6327±589 | 269±30 | 756±9 | 1029±165 | 21±3 | 60±13 | 748±46 | 13±2 | 4.6±0.8 | 7666±1427 | <9* | 2115±276 |
| **D3 48h 6m** | 7602±228 | 299±24 | 1135±88 | 866±154 | 15±4 | 105±25 | 858±46 | 17±3 | 4.3±1.0 | 9179±372 | <9* | 2105±201 |
| **D1 6h 1m** | 102±5 | 5.2±1.4 | 83±18 | 27±8 | 1.2±0.5 | 16±4 | 21±1 | <0.9* | 2.5±0.2 | na | <9* | 73±22 |
| **D1 24h 3m** | 96±10 | 4.1±0.7 | 36±3 | 18±2.5 | 0.6±0.3 | 5.1±0.7 | 18±2 | <0.9* | 2.2±0.2 | na | 17±9 | 61±3 |
| **D1 48h 6m** | 99±10 | 4.5±0.8 | 35±3 | 16±3 | <0.8* | 4.8±0.4 | 19±2 | <0.9* | 2.2±0.5 | na | <9* | 63±4 |
| **D3 7d 1m** | 3676±1070 | 266±50 | 89±29 | 973±288 | 7.9±3.1 | <0.9* | 443±115 | 1.5±0.1 | 4.6±1.4 | 7086±3278 | <9* | 2418±217 |
| **D3 7d 3m** | 6029±116 | 505±61 | 336±7 | 1404±200 | 20±1 | 10.3±1.8 | 391±49 | 8.9±1.5 | 6.7±1.4 | 7900±1549 | <9* | 4141±501 |
| **D3 7d 6m** | 4601±146 | 412±185 | 151±45 | 1927±200 | 31±1 | 5.7±0.9 | 419±32 | 3.6±0.7 | 8.4±0.7 | 7521±694 | <9* | 4384±93 |

**Table B.** Summary data on the occurrence and concentration (ng/L) of PPCP used in this study in European freshwaters (lakes and rivers). The table was drawn using the full Norman – Empodat database including records from monitoring activities conducted by tenths of European laboratories between 2005 and 2017. Norman is the Network of reference laboratories, research centres and related organisations for monitoring of emerging environmental substances (www.Norman-network.net ).

**Table C.** Nominal spiked concentrations (medium) and effective concentrations inhibiting 50% of growth (EC50) in phytoplankton species for the micropollutants composing the experimental mixture (expressed in ng / L, like in Table 1, main text). In the experiment, low and high corresponded to one tenth and ten times the levels reported for medium, respectively. Toxicity data were obtained from the U.S. Environmental Protection Agency ECOTOXicology Database System (2015, Version 4.0, [www.epa.gov/ecotox/](http://www.epa.gov/ecotox/)) when possible; if data were not available in the database, citations are reported for additional sources of information.

| **Chemical** | **CAS ID:** | **Therapeutic category** | **Spiked concentrations for medium** | **Mean EC50^§^** | **SD^§^** | **Num. of studies** |
| --- | --- | --- | --- | --- | --- | --- |
| atenolol | 29122-68-7 | anti-hypertensive | 1000 | 3.177E+08 | 2.629E+08 | 3 |
| bezafibrate | 41859-67-0 | lipid regulating | 100 | 3.497E+07 | 2.627E+07 | 3 |
| carbamazepine | 298-46-4 | anticonvulsant | 1000 | 1.368E+08 | 2.830E+08 | 24 |
| clarithromycin | 81103-11-9 | antibacterial | 1000 | 1.967E+04 | 2.325E+04 | 3^a^ |
| diclofenac | 15307-86-5 | antiinflammatory | 1000 | 6.268E+07 | 6.728E+07 | 6 |
| furosemide | 54-31-9 | diuretic | 100 | > 7.000E+07 | NA* | 1^b^ |
| hydrochlorothiazide | 58-93-5 | diuretic | 1000 | NA* | NA* | NA* |
| ibuprofen | 15687-27-1 | antiinflammatory | 100 | 3.286E+08 | 1.923E+07 | 2^c^ |
| ranitidine | 66357-35-5 | ulcer healing | 10 | 2.696E+07 | 4.872E+07 | 4^d^ |
| sulfamethoxazole | 723-46-6 | antibacterial | 10 | 2.149E+06 | 3.098E+06 | 7 |
| benzophenone-4 | 4065-45-6 | solar filter | 1000 | 1.000E+07 | NA* | 1 |
| triclosan | 3380-34-5 | antibacterial | 100 | 5.856E+05 | 7.823E+05 | 24 |

§ Average and standard deviation calculated for multiple species across the available studies; * NA = no data available / found; a) including also data from [18]; b) data from [19]; c) including also [20]; d) based on data on Rotifers, no toxicological data on algae were found.

**Table D.** Conditions used for analyses of the selected pharmaceuticals. The Turbo Ion Spray Source settings were: Ion Spray Voltage (IS) 5000V; Source Temperature 400°C; Nebulizer Gas (NEB) 8; Curtain Gas (CAD) 12; Collision Gas (CAD) 5.

| **Compounds** | **RT (min)** | **Source voltages**  **(V)** | | **Entrance Potential**  **(EP)** | **Precursor ion *(m/z)*** | **Product ion I *(m/z)* and collision energy (eV)** | **Product ion II *(m/z)* and collision energy (eV)** | **Collision Cell Exit Potential (CXP)** |
| --- | --- | --- | --- | --- | --- | --- | --- | --- |
|  |  | *Declustering potential (DP)* | *Focusing potential (FP)* |  |  |  |  |  |
| Atenolol | 3.8 | 30 | 180 | 10 | 267.1 | 190.1(26) | 145.1 (36) | 15 |
| Atenolol-d_7_ | 3.8 | 30 | 180 | 10 | 274.1 | 190.1 (27) | 145.1 (37) | 15 |
| Bezafibrate | 6.5 | -46 | -222 | -10 | 360.2 | 274.1 (-22) | 154.1 (-40) | -14 |
| Carbamazepine | 6.9 | 30 | 120 | 10 | 237.1 | 194.2 (28) | 192.1 (30) | 15 |
| Carbamazepine-d_10_ | 6.9 | 30 | 120 | 10 | 247.1 | 204.1 (28) | 201.1 (31) | 15 |
| Clarithromycin | 6.0 | 30 | 240 | 10 | 748.2 | 590.4 (26) | 158.1 (38) | 15 |
| Diclofenac | 7.3 | -32 | -200 | -10 | 294.1  296.1 | 250.1 (-16)  252.1 (-16) | -  - | -14 |
| Furosemide | 5.9 | -40 | -170 | -10 | 329.1 | 285.1 (-22) | 205.1 (-32) | -14 |
| Hydrochlorothiazide | 5.7 | -76 | -160 | -10 | 296.1 | 269.1 (-30) | 205.1 (-32) | -14 |
| Ibuprofen | 6.9 | -36 | -160 | -10 | 205.1 | 161.1 (-12) | - | -14 |
| Ibuprofen-d_3_ | 6.9 | -36 | -160 | -10 | 208.1 | 164.1 (-12) |  | -14 |
| Ranitidine | 4.0 | 31 | 120 | 10 | 315.2 | 176.1 (25) | 130.1 (35) | 15 |
| Sulfametoxazole | 4.7 | -36 | -200 | -10 | 252.1 | 156.1 (-22) | 92.1 (-38) | -14 |
| Benzophenone-4 | 5.4 | -40 | -200 | -10 | 307.1 | 211.1 (-35) | 227.1 (-33) | -14 |
| Triclosan | 8.0 | -30 | -120 | -10 | 289.1  287.1 | 35.1 (-35)  35.1 (-35) | -  - | -14 |

**Table E.** Factor loadings and principal components of Cytobuoy derived phytoplankton descriptors.

| **Parameter** | **PC1** | **PC2** | **PC3** |
| --- | --- | --- | --- |
| *Proportion of Variance* | *0.324* | *0.207* | *0.094* |
| *Cumulative Proportion* | *0.324* | *0.531* | *0.625* |
| Asymmetry.2.FL.Red | 0.007 | -0.082 | -0.012 |
| Asymmetry.FL.Orange | 0.076 | -0.059 | 0.217 |
| Asymmetry.FL.Red | 0.046 | -0.035 | 0.076 |
| Asymmetry.FL.Yellow | 0.078 | -0.055 | 0.199 |
| Asymmetry.FWS | 0.064 | -0.025 | 0.211 |
| Asymmetry.SWS | 0.085 | -0.014 | 0.211 |
| Average.2.FL.Red | 0.109 | -0.072 | -0.254 |
| Average.FL.Orange | 0.156 | -0.101 | -0.089 |
| Average.FL.Red | 0.089 | -0.108 | -0.246 |
| Average.FL.Yellow | 0.064 | -0.069 | -0.085 |
| Average.FWS | 0.124 | -0.130 | -0.174 |
| Average.SWS | 0.116 | -0.145 | 0.016 |
| Center.of.gravity.2.FL.Red | 0.223 | 0.053 | 0.056 |
| Center.of.gravity.FL.Orange | 0.222 | 0.050 | 0.082 |
| Center.of.gravity.FL.Red | 0.222 | 0.054 | 0.063 |
| Center.of.gravity.FL.Yellow | 0.222 | 0.052 | 0.078 |
| Center.of.gravity.FWS | 0.221 | 0.053 | 0.081 |
| Center.of.gravity.SWS | 0.220 | 0.055 | 0.087 |
| Fill.factor.2.FL.Red | -0.110 | 0.152 | 0.046 |
| Fill.factor.FL.Orange | -0.119 | 0.176 | -0.212 |
| Fill.factor.FL.Red | -0.093 | 0.171 | 0.006 |
| Fill.factor.FL.Yellow | -0.124 | 0.181 | -0.155 |
| Fill.factor.FWS | -0.053 | 0.183 | -0.251 |
| Fill.factor.SWS | -0.039 | 0.171 | -0.237 |
| Inertia.2.FL.Red | -0.075 | 0.166 | 0.139 |
| Inertia.FL.Orange | -0.069 | 0.231 | -0.121 |
| Inertia.FL.Red | -0.027 | 0.204 | 0.119 |
| Inertia.FL.Yellow | -0.079 | 0.222 | -0.054 |
| Inertia.FWS | 0.004 | 0.235 | -0.143 |
| Inertia.SWS | 0.033 | 0.229 | -0.119 |
| Length.2.FL.Red | 0.146 | 0.165 | 0.053 |
| Length.FL.Orange | 0.139 | 0.164 | -0.112 |
| Length.FL.Red | 0.143 | 0.165 | 0.015 |
| Length.FL.Yellow | 0.135 | 0.185 | -0.080 |
| Length.FWS | 0.170 | 0.135 | -0.096 |
| Length.SWS | 0.190 | 0.103 | -0.070 |
| Maximum.2.FL.Red | 0.131 | -0.088 | -0.218 |
| Maximum.FL.Orange | 0.172 | -0.077 | 0.043 |
| Maximum.FL.Red | 0.112 | -0.115 | -0.200 |
| Maximum.FL.Yellow | 0.100 | -0.083 | -0.055 |
| Maximum.FWS | 0.153 | -0.148 | -0.086 |
| Maximum.SWS | 0.135 | -0.147 | 0.094 |
| Number.of.cells.2.FL.Red | 0.106 | 0.173 | 0.188 |
| Number.of.cells.FL.Orange | 0.122 | 0.207 | 0.057 |
| Number.of.cells.FL.Red | 0.128 | 0.171 | 0.163 |
| Number.of.cells.FL.Yellow | 0.069 | 0.237 | 0.018 |
| Number.of.cells.FWS | 0.162 | 0.167 | 0.075 |
| Number.of.cells.SWS | 0.177 | 0.151 | 0.063 |
| Total.2.FL.Red | 0.161 | -0.028 | -0.201 |
| Total.FL.Orange | 0.158 | -0.026 | -0.047 |
| Total.FL.Red | 0.141 | -0.060 | -0.218 |
| Total.FL.Yellow | 0.156 | -0.044 | -0.099 |
| Total.FWS | 0.169 | -0.032 | -0.131 |
| Total.SWS | 0.216 | -0.033 | -0.006 |

**Table F.** Spearman correlation of concentration levels (non-parametric) computed among all chemical compounds (without initial levels) in our experimental microcosms, text in red indicates a significant correlation (p < 0,05).


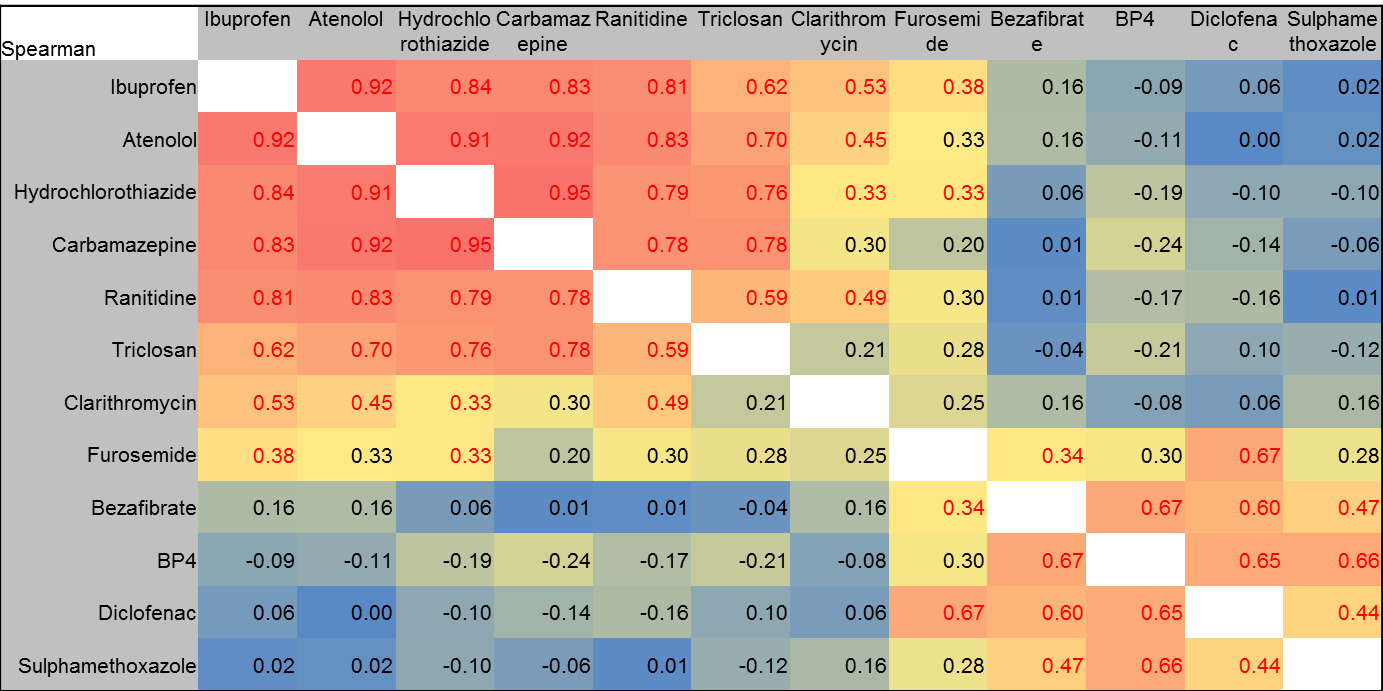


| ** | p < 0.05 |
| --- | --- |
| * | 0.05 < p <= 0.10 |
| - | p > 0.10 |

**Table G.** Test among depths for concentrations of micropollutants - Kruskal-Wallis test (nonparametric).


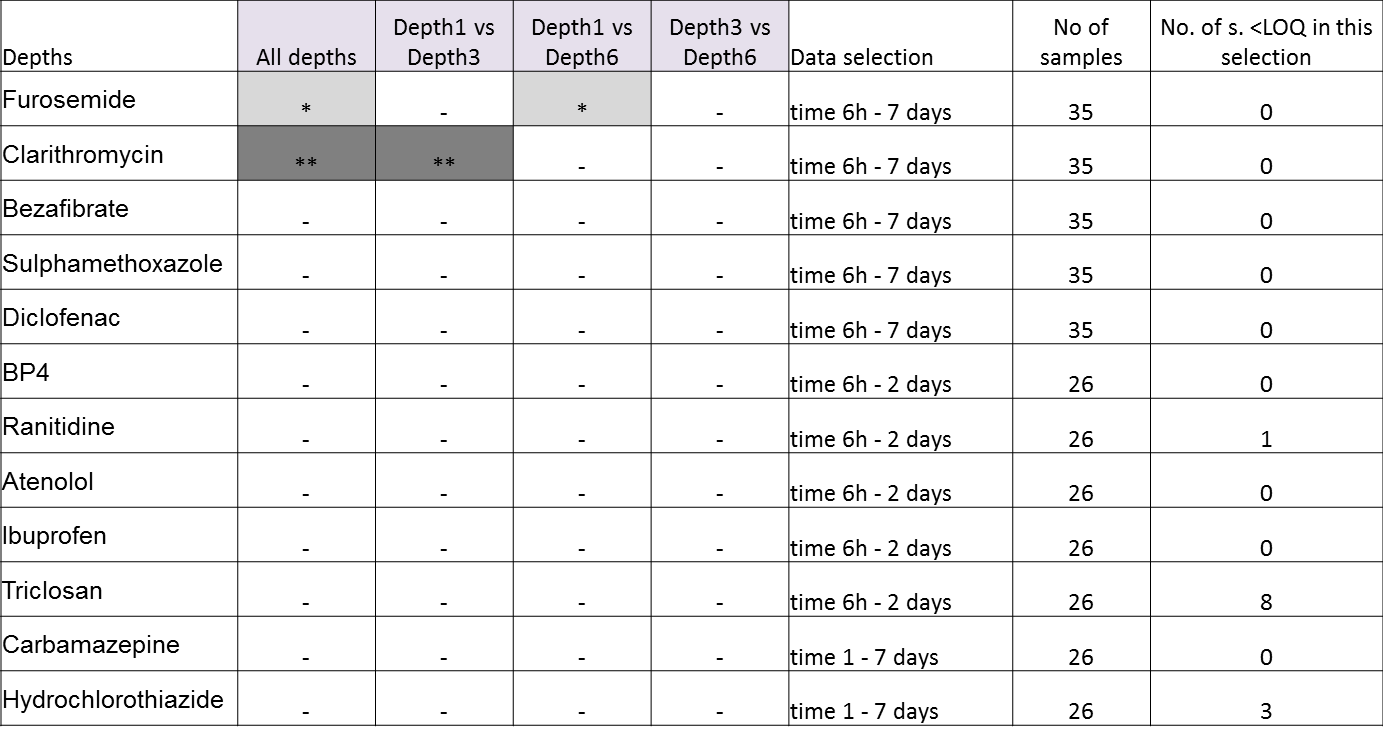


**Table H.** Results of GLM for estimation of factor significance, direction and effects (partial Eta-squared) in experimental communities. Residuals of GLM for bacterial cells concentration were not significant for the reported response variables and therefore are omitted from this table. Similarly, intercepts from GLM are not reported. Statistically significant effects at p < 0.05 are highlighted as grey cells, light grey highlights p < 0.1.

|  |  |  | **Factors** | | | | |
| --- | --- | --- | --- | --- | --- | --- | --- |
|  |  |  | **random^(a)^** | **depth** | **dose** | **interaction** | **initial levels** |
| **PC1 (pigment packaging, length)** | *Average* | *p*-value | 0.000 | 0.003 | 0.070 | 0.140 | 0.635 |
|  |  | Partial Eta^2^ | 0.826 | 0.265 | 0.175 | 0.231 | 0.005 |
|  |  | Direction | NA | - | + | - | + |
|  | *Variance* | *p*-value | 0.061 | 0.000 | 0.013 | 0.011 | 0.669 |
|  |  | Partial Eta^2^ | 0.547 | 0.399 | 0.263 | 0.357 | 0.004 |
|  |  | Direction | NA | - | - | - | + |
| **PC2 (scattering and shape)** | *Average* | *p*-value | 0.000 | 0.026 | 0.157 | 0.046 | 0.783 |
|  |  | Partial Eta^2^ | 0.850 | 0.189 | 0.134 | 0.293 | 0.002 |
|  |  | Direction | NA | + | + | - | + |
|  | *Variance* | *p*-value | 0.000 | 0.262 | 0.024 | 0.430 | 0.534 |
|  |  | Partial Eta^2^ | 0.902 | 0.072 | 0.233 | 0.148 | 0.008 |
|  |  | Direction | NA | - | - | + | - |
| **Cells mL^-1^** | *Total* | *p*-value | 0.000 | 0.000 | 0.000 | 0.029 | 0.931 |
|  |  | Partial Eta^2^ | 0.898 | 0.454 | 0.724 | 0.316 | 0.000 |
|  |  | Direction | NA | + | + | - | + |
| **Biomass (µg L^-1^)** | *Total* | *p*-value | 0.000 | 0.003 | 0.000 | 0.269 | 0.444 |
|  |  | Partial Eta^2^ | 0.894 | 0.284 | 0.677 | 0.186 | 0.013 |
|  |  | Direction | NA | + | + | - | + |
| **Phenotypic diversity** | *Total* | *p*-value | 0.021 | 0.000 | 0.000 | 0.048 | 0.391 |
|  |  | Partial Eta^2^ | 0.585 | 0.403 | 0.427 | 0.282 | 0.016 |
|  |  | Direction | NA | - | - | - | + |
| **Length (µm)** | *Average* | *p*-value | 0.000 | 0.255 | 0.317 | 0.145 | 0.959 |
|  |  | Partial Eta^2^ | 0.780 | 0.072 | 0.093 | 0.227 | 0.000 |
|  |  | Direction | NA | + | + | - | + |
|  | *Variance* | *p*-value | 0.000 | 0.001 | 0.027 | 0.056 | 0.335 |
|  |  | Partial Eta^2^ | 0.705 | 0.316 | 0.225 | 0.282 | 0.020 |
|  |  | Direction | NA | - | - | - | - |
| **Number of cells per colony** | *Average* | *p*-value | 0.000 | 0.687 | 0.342 | 0.068 | 0.569 |
|  |  | Partial Eta^2^ | 0.831 | 0.020 | 0.088 | 0.273 | 0.007 |
|  |  | Direction | NA | + | + | - | - |
|  | *Variance* | *p*-value | 0.000 | 0.032 | 0.026 | 0.081 | 0.544 |
|  |  | Partial Eta^2^ | 0.821 | 0.179 | 0.222 | 0.259 | 0.008 |
|  |  | Direction | NA | - | - | + | - |
| **Particle biomass (µg)** | *Average* | *p*-value | 0.000 | 0.014 | 0.266 | 0.235 | 0.429 |
|  |  | Partial Eta^2^ | 0.857 | 0.204 | 0.101 | 0.195 | 0.013 |
|  |  | Direction | NA | - | + | - | + |
|  | *Variance* | *p*-value | 0.007 | 0.004 | 0.016 | 0.636 | 0.858 |
|  |  | Partial Eta^2^ | 0.616 | 0.266 | 0.253 | 0.109 | 0.001 |
|  |  | Direction | NA | - | - | + | + |
| **Total Chl-a FL** | *Total* | *p*-value | 0.000 | 0.050 | 0.005 | 0.003 | 0.247 |
|  |  | Partial Eta^2^ | 0.870 | 0.156 | 0.301 | 0.409 | 0.028 |
|  |  | Direction | NA | + | + | - | + |
| **Bacterial cells (µL^-1^)** | *Total* | *p*-value | 0.002 | 0.019 | 0.000 | 0.000 | 0.000 |
|  |  | Partial Eta^2^ | 0.166 | 0.096 | 0.686 | 0.343 | 0.234 |
|  |  | Direction | NA | + | + | - | - |

(a) experimental bags were considered in the DOE analysis as random factors nested within the interaction between dose and depth.

**
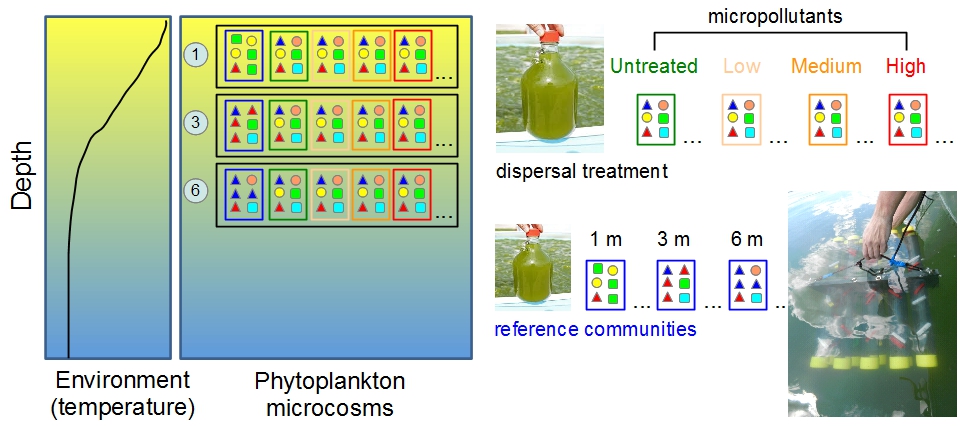
Fig. A.** **Panel** **(A)** Cartoon of the experimental set up. Coloured shapes inside boxes (microcosms) represent different trait combinations. Black boxes represent racks deployed at different depths (1, 3 and 6 m). The photograph depicts an experimental rack filled with bag microcosms during deployment (a movie about deployed racks and microcosms is also available at <http://youtu.be/cTT9NaFx63k>). **Panel** **(B)** Schematic representation of the procedure to test for deviation of community metrics from random assembly of initial individuals: *n* individuals were randomly sampled from the *m* staring pool, and community metrics were calculated for 100 times. The final observed datum in the focal metric was ranked in the random distribution of expected values, and the rank position saved.

**A)**


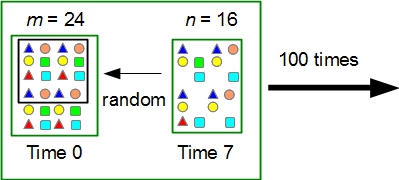
**B)**


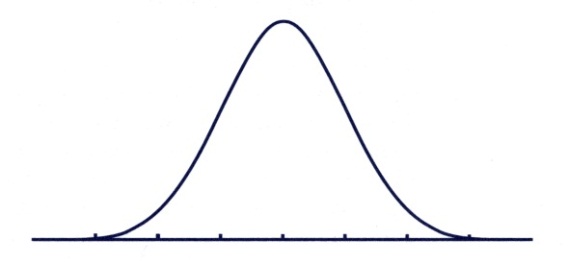


frequency

normalized value

observed datum

(end of experiment)

**Fig. B.** Time trends and associated regression models derived from experimental data (high concentration samples) for the chemicals composing the studied mixture.

**Fig. C.** Average values of phytoplankton individual-level trait data. Average of PC1 (A - eigenvalue) and PC2 (B- eigenvalue) (for trait variance explained and factor loadings see Table S4), and average length (C- µm), number of cells (D) and biomass (E- ng) of individual particles in local reference communities and experimental mixed communities. Points represent mean ± standard error.

**Fig. D.** Variance of phytoplankton individual-level trait data: PC1 (A) and PC2 (B) (see Table S4), length (C), number of cells (D) and biomass (E) of individual particles in local reference communities and experimental mixed communities. Points represent mean ± standard error.

**Fig. E.** Phytoplankton overall community data. Phenotypic diversity (A), total community biomass per unit of volume (B- µg/L), total biomass per unit of functional diversity (C), phytoplankton cells concentration (D- cells/mL) and total Chl-a fluorescence for all particles (E – Log[mV]) in local reference communities and experimental mixed communities. Points represent mean ± standard error.

**Fig. F.** Characterisation of environmental changes in lake Greifensee during the experiment. Meteorological conditions: air temperature (A) and rain accumulation (B); the vertical red lines indicate the experimental period of time. Physico-chemical data at experimental depths (time 0 and time 7 correspond to the 27^th^ of June and the 4^th^ of July 2012, respectively – the red lines in A-B): C-G) depth plots of water temperature (°C), conductivity (µS), pH, N-NO3 (mg/L) and P-PO4 (µg/L), respectively; H) cluster dendrogram (Euclidean distance, average linkage) of depths based on water environmental variables.

**Fig. G.** Effects of depth and micropollutants on deviation of local reference communities and experimental mixed communities from random assembly (points represent mean ± standard error): A) total Chl-a fluorescence, B) average length of particles, C) variance in length, D) average biomass of particles, E) variance in biomass, F) average number of cells per particle, G) variance in number of cells per particle. X-axis represents the rank position of final community values in the distribution of expected values from random draws of individuals from initial communities.

**Fig. H.** Treatment with different initial spikes generated different exposure scenarios in terms of duration and composition of the mixture (A-C). The most persistent pollutants are the same in the three exposure scenarios. Trends were modelled by regression from experimental data (see Fig. S2). In this figure, lake background levels of pollutants were subtracted from model predictions, and panels A-C characterise exposure scenarios above the background contamination.
